# Hunger and microbiology: is a low gastric acid‐induced bacterial overgrowth in the small intestine a contributor to malnutrition in developing countries?

**DOI:** 10.1111/1751-7915.12780

**Published:** 2017-07-17

**Authors:** Shafiqul A. Sarker, Tahmeed Ahmed, Harald Brüssow

**Affiliations:** ^1^ Nutrition and Clinical Services Division International Centre for Diarrhoeal Disease Research Dhaka Bangladesh; ^2^ Nutrition and Health Institute Gut Ecosystem Department Host‐ Microbe Interaction Group Nestlé Research Centre CH‐1000 Lausanne 26 Switzerland

## Abstract

Underproduction of hydrochloric acid into the stomach is frequently encountered in subjects from developing countries. We explore the hypothesis that hypochlorhydria compromises the gastric barrier and favours bacterial overgrowth in the proximal parts of the small intestine where nutrient absorption takes place. Food calories are thus deviated into bacterial metabolism. In addition to an adequate caloric supply, correcting hypochlorhydria might be needed to decrease childhood malnutrition.

## Introduction

The United Nations (UN) have formulated an action plan for sustainable development, of which the two top‐ranking goals for 2030 are to end both poverty and hunger. To improve nutrition, the UN wants to achieve food security and promote sustainable agriculture. It is undisputable that eating too few calories leads over time to undernutrition. However, food security might be necessary, yet insufficient alone to correct malnutrition, as other factors are proposed to contribute. For example, malnourished children are frequently found side by side with non‐malnourished children in field studies, and nutritionists underline difficulties in correcting malnutrition by high‐calorie food alone (Ahmed *et al*., 2009). A landmark study demonstrated that refeeding caused better weight gain when combined with antibiotics, therefore pointing to a role of microbes in malnutrition (Trehan *et al*., 2013). The idea of using antibiotics in malnutrition has been and continues to be controversially discussed for now over 50 years (Alcoba *et al*., 2013; Brüssow, 2015), and an impact of the gut microbiota on human nutrition is not farfetched (Blanton *et al*., 2016). By fermenting complex polysaccharides that are inaccessible to human digestive enzymes, the gut microbiota releases short‐chain fatty acids (SCFAs) as metabolic end‐products (Sonnenburg *et al*., 2005), which are avidly taken up systemically and by the colon enterocytes and contribute thus to human nutrition and have important signalling functions (Brüssow and Parkinson, 2014). J. Gordon's laboratory pioneered the idea that the composition of the gut microbiota determined the extent of this extra calorie contribution and associated a particular microbial community characterized in first approximation by the ratio of Firmicutes to Bacteroidetes with obesity (Ley *et al*., 2006). When the microbiota–obesity connection turned out to be more complicated than initially thought, J. Gordon's laboratory teamed up with nutritionists to study the effect of gut microbiota composition on malnutrition. However, twin studies from Africa showed no remarkable differences in overall gut microbiota composition between children with kwashiorkor (i.e. protein malnutrition) and their nutritionally normal siblings. Only when their stool microbiota was transferred into axenic mice did differences with respect to energy extraction become detectable when mice were also fed local diets from African children (Smith *et al*., 2013). Malnourished children from Bangladesh showed a delayed gut microbiota maturation compared with age‐matched healthy children, but otherwise no gross faecal microbiota dysbiosis (Subramanian *et al*., 2014). Yet, the gut microbiota of the large intestine is only part of the microbiota–malnutrition equation, and it is possible that microbes colonizing the upper parts of the small intestine have a potentially larger impact on malnutrition than those colonizing the colon.

**Figure 1:**
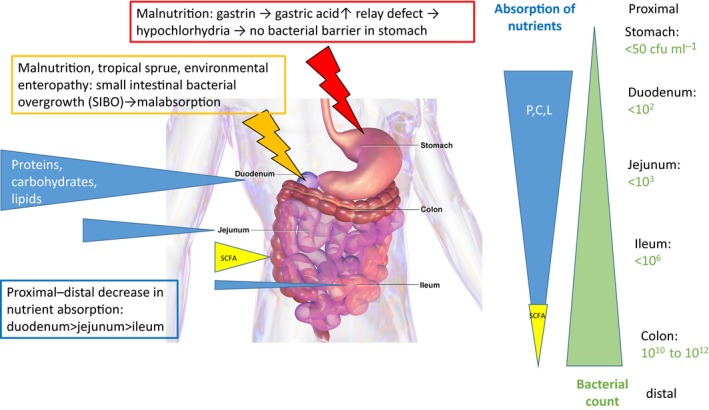
The antiparallel gradient of absorption capacities (blue wedges, size is proportional to absorption capacity) for proteins (P), carbohydrates (C) and lipids (L) compared to bacterial colonization (green wedge, size is proportional to titres indicated on right) along the proximal‐to‐distal axis of the human gastrointestinal tract, with the notable exception of short‐chain fatty acid (SCFA) absorption (yellow wedge) in the colon. Data from Gorbach *et al*. (1967). Broken arrows indicate two pathological conditions that interfere with the distribution of bacterial colonization along the gastrointestinal tract: low gastric acid production (red symbol) and bacterial overgrowth (orange symbol) in the upper small intestine. The background anatomical gut picture is from Blausen.com staff *Wikiversity Journal of Medicine*.

The argument runs as follows: in the upper parts of the alimentary tract, there is a conspicuous antiparallel distribution of absorptive capacities for the major three classes of caloric nutrients (proteins, carbohydrates, fats) and gut colonization with bacteria (Fig. 1; Brüssow, 2007). Digestive enzymes for carbohydrates are already produced into the oral cavity. In response to food powerful proteases (pepsin) are secreted into the acidic environment of the stomach. The exocrine pancreas then secretes the bulk of carbohydrate‐ and lipid‐digesting enzymes and a second wave of proteolytic enzymes (trypsin, chymotrypsin) into the duodenum. The absorption of nutrients is minimal from mouth to stomach, while the major share of protein, carbohydrate and lipid digestion products is absorbed in the duodenum and the upper parts of the jejunum, smaller amounts are absorbed in the lower parts of the jejunum and even less in the ileum. This proximal‐to‐distal decline in absorption function along the human gut finds a mirror image in the level of bacterial colonization (Fig. 1). The duodenum is only sparsely colonized with < 100 bacteria per ml aspirate. Bacterial titres are increasing further down to reach levels of 10^6^ bacteria per ml aspirate in the distal ileum. The ileocaecal valve marks the transition from the small into large intestine and the transition to a high bacterial load in the colon with 10^12^ bacteria per gram gut content (Roland *et al*., 2014). From a physiological and an evolutionary viewpoint, this reciprocal distribution of absorption and bacterial colonization makes sense. The digestion of food by host enzymes transforms nutrients into absorbable forms that are then transported across the gut epithelium into lymph and blood stream. The presence of high titres of gut bacteria in the first 50 cm of the small intestine would represent undesirable food competitors stealing calories and diverting nutrients from host metabolism into bacterial biomass production. This would therefore reduce the efficiency of calorie extraction from food, which might become critical for the host if the food supply is limiting.

Keeping food competitors in check is, in energy terms, costly, but the control of undesired bacterial colonization is among other mechanisms (e.g. secretion of antimicrobial peptides by the gut) achieved by two major mechanisms that are at the same time necessary physiological requirements for food processing to gain energy and nutrients. The first such energy‐costing barrier against bacterial competitors in the absorptive sections of the gut is acid production in the stomach, which is also needed for efficient protein digestion by pepsin. Hydrochloric acid is an efficient penetration barrier for bacteria accompanying food or being swallowed with highly colonized oral secretions. When gastric acidity drops to pH 3, a rapid bactericidal effect was documented for gastric juice *in vitro* and *in vivo*, which was not observed at pH 4 (Giannella *et al*., 1972). Children produce less gastric acidity than adults (milk proteins are easier digested than meat), but the mean resting gastric pH remains below 3 (Maffei and Nóbrega, 1975). The second barrier function against bacterial overgrowth in the small intestine is gut peristalsis, which mixes and pushes the gut content forward for further processing, thus representing another physiologically needed energy investment. Towards the terminal ileum, peristalsis becomes less vigorous and, concomitantly, bacterial titres increase. The antibacterial efficiency of these two mechanisms has been demonstrated by clinical observations. Drugs that suppress gastric acid secretion (omeprazole) cause bacterial overgrowth in the duodenum (> 10^5^ cfu ml^−1^) in half of the patients (Fried *et al*., 1994; Thorens *et al*., 1996). Bacterial overgrowth in the small intestine, as assessed by hydrogen breath tests, was also associated with disorders of peristalsis (Vantrappen *et al*., 1977; Roland *et al*., 2015).

When one of these two major barrier functions break down, the body opens gates for an increased bacterial colonization of the gut. Fifty years ago, gastroenterologists reported that malnourished children showed basal gastric acid output below normal (‘hypochlorhydria’). Also, maximal acid output after stimulation by subcutaneous pentagastrin was reduced in 50% of subjects (Gracey *et al*., 1977). About half of malnourished children from Bangladesh and Brazil showed gastric pH above 4 and even after nutritional rehabilitation, the rates of hypochlorhydria remained unchanged (Maffei and Nóbrega, 1975; Gilman *et al*., 1988), therefore excluding acute energy deficiency as a cause for this condition. Hypochlorhydria is widely distributed in malnourished children and has also been described in Indonesia, Nigeria and South Africa (Sarker and Gyr, 1992). At the same time, several studies have described high titres of microbes in the upper small intestine of malnourished children, with a mean titre of 5 × 10^6^ viable bacteria per mL jejunal fluid in Gambia (Heyworth and Brown, 1975) and Latin America (Mata *et al*., 1972) and mean titres of 8 × 10^7^ ml^−1^ in Indonesia (Gracey *et al*., 1973); some children showed > 10^10^ bacteria per ml (Heyworth and Brown, 1975). Most of these children also showed chronic diarrhoea. Paediatricians from Nigeria found comparable bacterial counts of about 10^7^ ml^−1^ duodenal aspirate in malnourished children with and without diarrhoea, while well‐nourished children with and without diarrhoea both displayed < 10^5^ cfu ml^−1^ intestinal aspirate (Omoike and Abiodun, 1989). A Brazilian study showed that the gastric count of coliform bacteria in malnourished children was proportional to the gastric pH, closely linking both phenomena (Maffei and Nóbrega, 1975).

With these bacterial titres at sites critical for caloric nutrient absorption, a food drain can be anticipated, but has yet to be experimentally proven. In addition, bacterial metabolites and endotoxins released from decaying bacteria might harm the mucosa and cause inflammation, while bacterial‐mediated deconjugation of bile salts (Cassells *et al*., 1970) could lead to problems with fat absorption.

A number of observations suggest that the problem of low gastric acid production is widespread in developing countries (Sarker and Gyr, 1992), while the full extent is currently still unknown. More than half of better‐nourished Bangladeshi children also showed a basal gastric pH ≥ 4, but experienced a drop in pH upon betazole (histamine H2 receptor agonist) stimulation (Gilman *et al*., 1988). None of them showed bacterial overgrowth, pointing to the importance of pH in the digestive phase for barrier function. Forty per cent of Bangladeshi cholera patients presented with hypochlorhydria, which was identified as a risk factor and not as a consequence of cholera (Nalin *et al*., 1978). Stimulated acid production was also significantly lower in healthy controls from lower socio‐economic groups than from higher ones in Indian men (Sack *et al*., 1972). This observation concurs with epidemiology data on cholera susceptibility and points to an environmental factor that influences gastric acid production in developing countries.

Small intestinal bacterial overgrowth (SIBO) was also reported in tropical sprue (Gorbach *et al*., 1970; Ghoshal *et al*., 2003), an enigmatic enteropathy of tropical countries with suspected, but not proven, infectious aetiology characterized by malabsorption symptoms (Ghoshal *et al*., 2014; Wanke, 2014). However, overgrowth was not a consistent observation in tropical sprue (Bhat *et al*., 1972) and the pathology seems to correlate more closely with enterotoxin levels (Klipstein *et al*., 1978) than the sheer amount of bacteria in the small intestine. For ethical reasons, SIBO diagnosis by gastric and intestinal intubation has been largely replaced with a non‐invasive hydrogen breath test. Notably, this breath test diagnosed SIBO in > 30% of children from urban slums in Brazil compared to 2% SIBO diagnosis in children from the same city attending private schools (dos Reis *et al*., 2007; Mello *et al*., 2012). As expected, both groups differed for anthropometric measures and environmental hygiene levels, while no difference was seen for these parameters between slum dwellers with or without SIBO diagnosis. Breath tests returned to normal after antibiotic treatment (Tahan *et al*., 2013). A prospective study from an impoverished Bangladeshi neighbourhood revealed 17% of children with SIBO and SIBO correlated with stunting and open sewers (Donowitz *et al*., 2016). This apparently rather widespread condition (Donowitz and Petri, 2015) is linked with environmental enteropathy (EE). The relationship of EE with tropical sprue is controversial, and while its pathogenesis is poorly understood, it includes chronic intestinal inflammation (Donowitz *et al*., 2016). Whether SIBO in EE is associated with hypochlorhydria, which also showed an association with low socio‐economic status, is not clear at present.

Environmental enteropathy is currently an active research area, and treatment modalities for EE are at the moment not clear. Its underlying pathology might explain the failure of isolated nutritional interventions in correcting malnutrition and growth delays. Controlled studies of dietary supplements with antibiotics, limited to a crucial infantile growth phase, have been proposed in the past (Gorbach, 1972) and antibiotics are indeed effective in tropical sprue. Hypochlorhydria has additionally been linked with *Helicobacter pylori* infection in developing countries, and gastric acid secretion ameliorated with the eradication of this pathogen (Sarker *et al*., 2004, 2012). However, such an intervention would require antibiotic treatment, which is difficult to envision as a mass application in view of the high prevalence of *H. pylori* infection in developing countries (Mahalanabis *et al*., 1996) in conjunction with antibiotic resistance problems. Some observations from the literature might provide hints for treatment options. Half of breastfed children from a Brazilian study showed a resting pH above 4; some showed elevated coliform counts in the stomach, while others did not (Maffei and Nóbrega, 1975). Apparently, some mothers provide breast milk that contains compounds (oligosaccharides?) that protect against bacterial overgrowth of the upper gut in the absence of a gastric barrier. Alternative pharmacological approaches should also be explored to address the fact that impaired acid production by gastric parietal cells upon gastrin and histamine H2 receptor agonist stimulation is a consistent observation in malnourished children. Some beverages are known to stimulate gastric acidity production, like coffee, alcoholic beverages and particularly digestive bitters, whose bitter ingredients also exist as a phytochemical (Tinctura amara) for hypochlorhydria treatment (Lüllmann, 2016). Finally, one could also envision direct acidification of the stomach with a pharmaceutical preparation of citric acid used in hypo‐ and achlorhydria patients (Lüllmann, 2016) or local low pH beverages.

In summary, to reach the UN sustainability goals with respect to improved nutrition in developing countries, achieving food security is not enough. The problem of low gastric acidity should also be addressed as it compromises protein digestion, leads to the production of possibly carcinogenic nitrosamine compounds via intragastric colonization (Wang *et al*., 2014) and limits the absorption of iron (gastric acid converts ferric iron to its absorbable ferrous form), leading to widespread iron deficiency anaemia in developing countries (Sarker *et al*., 2004). As early colonization with *H. pylori* may contribute to atrophic gastritis and thereby to low gastric acid production (El‐Omar *et al*., 1997), sanitation programs to decrease *H. pylori* transmission via drinking water (Klein *et al*., 1991) should also be considered. As the strength of any causal relationship for any linked observation increases from association studies to prospective studies and finally intervention studies, the link between insufficient gastric acid production and malnutrition is best tested by assessing the impact of citric acid supplementation in malnourished subjects during a refeeding trial.

## Conflict of interest

None declared.
